# Supplementary material for: South African university students’ experiences of online group cognitive behavioural therapy: Implications for delivering digital mental health interventions to young people
Source: Glob Ment Health (Camb). 2023 Jul 28;10:e45. doi: 10.1017/gmh.2023.39 (PMC10579664; doi:10.1017/gmh.2023.39)
Supplement: Supplementary file 1 [file S2054425123000390sup001.docx]

**Interview schedule for individual interviews**

Can you tell us about your experience of using the psychological skills training group intervention?

What did you like about it?

What didn’t you like about it?

What made it easy / difficult to engage with?

How does this intervention compare with any other experiences you have had of mental health treatments / counselling?

Would you recommend the intervention to other people?

Why / why not?

Whom (what kinds of people) do you think would like this kind of intervention?

What suggestions would you have to make the intervention more appealing to SA students?

Content?

Presentation of information?

Format?

How can the application be made more culturally appropriate for use among SA students?

Do you have any other comments or suggestions about the intervention?
